# ViraLM: empowering virus discovery through the genome foundation model

**DOI:** 10.1093/bioinformatics/btae704

**Published:** 2024-11-23

**Authors:** Cheng Peng, Jiayu Shang, Jiaojiao Guan, Donglin Wang, Yanni Sun

**Affiliations:** Department of Electrical Engineering, City University of Hong Kong, Hong Kong (SAR), China; Department of Information Engineering, The Chinese University of Hong Kong, Hong Kong (SAR), China; Department of Electrical Engineering, City University of Hong Kong, Hong Kong (SAR), China; School of Environmental Science and Engineering, Shandong University, Qingdao 266200, China; Department of Electrical Engineering, City University of Hong Kong, Hong Kong (SAR), China

## Abstract

**Motivation:**

Viruses, with their ubiquitous presence and high diversity, play pivotal roles in ecological systems and public health. Accurate identification of viruses in various ecosystems is essential for comprehending their variety and assessing their ecological influence. Metagenomic sequencing has become a major strategy to survey the viruses in various ecosystems. However, accurate and comprehensive virus detection in metagenomic data remains difficult. Limited reference sequences prevent alignment-based methods from identifying novel viruses. Machine learning-based tools are more promising in novel virus detection but often miss short viral contigs, which are abundant in typical metagenomic data. The inconsistency in virus search results produced by available tools further highlights the urgent need for a more robust tool for virus identification.

**Results:**

In this work, we develop ViraLM for identifying novel viral contigs in metagenomic data. By using the latest genome foundation model as the backbone and training on a rigorously constructed dataset, the model is able to distinguish viruses from other organisms based on the learned genomic characteristics. We thoroughly tested ViraLM on multiple datasets and the experimental results show that ViraLM outperforms available tools in different scenarios. In particular, ViraLM improves the F1-score on short contigs by 22%.

**Availability and implementation:**

The source code of ViraLM is available via: https://github.com/ChengPENG-wolf/ViraLM.

## 1 Introduction

As the most abundant biological entities on Earth, viruses play significant roles in regulating microbial communities, mediating horizontal gene transfer, and impacting public health. Although significant progress has been made in studying viruses across various ecosystems, our understanding of the diversity of viruses is still limited. It is estimated that the total number of distinct virus species is about 10^7^–10^9^, far exceeding the number of viruses currently cataloged in the viral genome databases (Koonin *et al.* 2023). Recently, the advent of high-throughput sequencing enables researchers to directly sequence all the genetic materials from various samples, leading to a wealth of metagenomic sequencing data. Consequently, mining viral sequences from metagenomic data, which also encompasses genetic materials from bacteria, archaea, and even eukaryotes, has become a critical step in exploring the full diversity of viruses.

Several attempts have been made to tackle the virus identification task, most of which incorporate machine learning to address the limitation of alignment-based methods which fail to identify novel viruses that do not possess significant similarities with the reference genomes. These methods can be roughly categorized into two types based on the major adopted features: nucleotide-based (Ren *et al.* 2020, Dong *et al.* 2024) and protein-based (Kieft *et al.* 2020, Guo *et al.* 2021, Camargo *et al.* 2023, Rangel-Pineros *et al.* 2023). Despite the promising results, methods that rely on proteins typically require the presence of relatively complete protein organization in query sequences. This limitation restricts their ability to accurately predict sequences with few proteins or sequences derived from noncoding regions.

In this study, we introduce Viral Language Model (ViraLM), a virus identification tool powered by a pre-trained general-purpose genome foundation model. ViraLM uses the latest genome foundation model DNABERT-2 (Zhou *et al.* 2023), which was pre-trained on a vast array of organisms, as the backbone to acquire valuable representations of DNA sequences. These representations enable ViraLM to effectively distinguish viral sequences from nonviral ones. In addition, we constructed a substantial virus dataset comprising 49 929 high-quality viral genomes spanning diverse taxonomic groups as positive samples and a negative training set including sequences commonly misclassified as viruses, such as prokaryotic host genomes (bacteria and archaea) and eukaryotes that are frequent virus hosts or vectors, like insects. We extensively tested ViraLM on several datasets, including the RefSeq benchmark dataset, the IMG/VR dataset, and three real metagenomic data. We benchmarked ViraLM with four leading protein-based tools [geNomad (Camargo *et al.* 2023), VIRify (Rangel-Pineros *et al.* 2023), VirSorter2 (Guo *et al.* 2021), and VIBRANT (Kieft *et al.* 2020)] and two nucleotide-based tools [VirRep (Dong *et al.* 2024) and DeepVirFinder (Ren *et al.* 2020)]. Our experimental results show that ViraLM consistently outperforms these existing tools in various experiments, with a notable 22% improvement in the F1-score for short contigs.

## 2 Methods and materials

**Figure 1.**
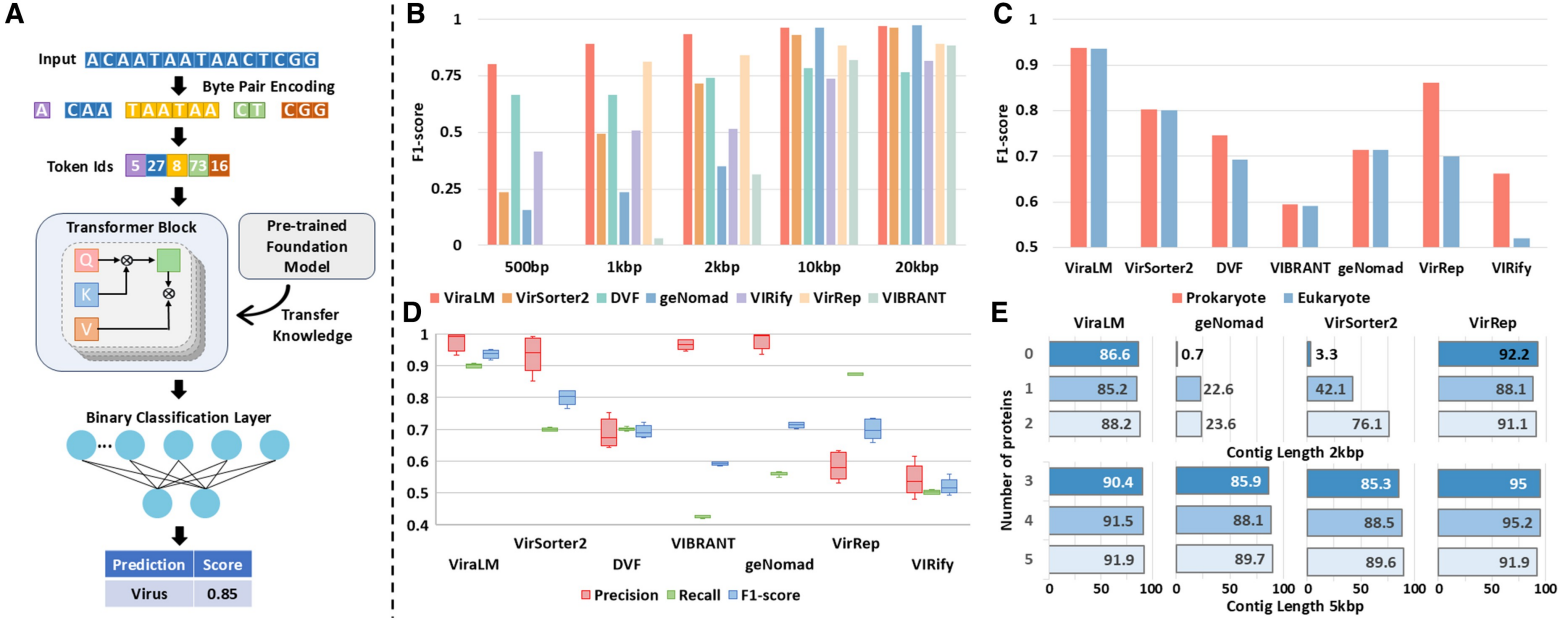
(A) The framework of ViraLM. The input sequence is tokenized and fed into the transformer block. Then the binary classification layer will aggregate the result from the transformer block to generate a final prediction. (B) The performance of each tool on various-length contigs where negative samples only consist of prokaryotes (bacteria, archaea, plasmid). (C) Comparison of the performances on prokaryotic (bacteria, archaea, plasmid) and eukaryotic (fungi, protozoa, insects, bats, and humans) genomes. (D) The performance of each tool on distinguishing viruses from eukaryotic contigs (fungi, protozoa, insects, bats, and humans). (E) Sensitivity of virus identification on contigs with various protein densities, grouped by contig lengths. *X*-axis: percentage of identified viruses (sensitivity). *Y*-axis: number of proteins.

The framework of ViraLM is illustrated in Fig. 1A. The main architecture of ViraLM contains three parts: a Byte-Pair Encoding block, a multi-layer Transformer block, and a specific binary classifier layer for virus classification (detailed in Supplementary Section S1.1). Utilizing the knowledge from pre-trained DNABERT-2 yields two main benefits for ViraLM. First, the self-supervised learning approach used in DNABERT-2 allows ViraLM to leverage genomic data without the need for laborious labeling. This overcomes the limitations of task-specific supervised learning, which relies on limited labeled datasets. Second, by initializing ViraLM with the knowledge (parameters) from DNABERT-2, the model can rapidly converge in the virus identification task through fine-tuning, resulting in accelerated training and prediction. In the prediction process, the input sequence is automatically segmented into nonoverlapping 2 kb fragments, which are then tokenized and fed into the Transformer block. Each fragment has a prediction score (between 0 and 1.0), indicating the likelihood of the input fragment being a viral sequence. The prediction scores of the segments from the same sequence are averaged to generate a final prediction. By default, an input sequence with a final prediction score over 0.5 will be classified as a viral sequence.

In the following section, we first elaborate on how we transfer and tailor the prior knowledge learned by the pre-trained foundation model for the virus identification task. Then, we will detail the process of constructing the comprehensive training and test datasets for our experiments.

### 2.1 Transfer knowledge from pre-trained foundation model

Recent research has shown that machine learning models trained on diverse, multi-species genomic data tend to outperform those trained on single-species data (Dalla-Torre *et al.* 2023). This underscores the importance of using a large and diverse dataset to enhance a model’s learning capacity. However, the existing methods often suffer from the limited number of high-quality task-specific labeled data, which can hinder their ability to effectively extract the most informative patterns.

To address the limitation, we incorporate prior knowledge from the pre-trained foundation model DNABERT-2, which is pre-trained on an expansive dataset containing 32.5 billion nucleotide bases, sourced from the genomes of 135 different species across seven diverse categories, including bacteria, fungi, protozoa, mammals, and other vertebrates. This pre-training utilizes Masked Language Modeling (MLM) (Devlin *et al.* 2018), a self-supervised learning strategy that enables the model to discern general patterns and motifs from the unlabeled data, independent of any specific task. During pre-training, approximately 15% of the input tokens are randomly selected and replaced (masked) with either a specific [*MASK*] token or a randomly chosen different token. Then the model is tasked with predicting these masked tokens based on their surrounding context. By using MLM, DNABERT-2 gains the ability to comprehend the various contexts in which each nucleotide base appears, thus enabling a comprehensive understanding of genomic sequences.

A transfer and fine-tuning method is applied to combine the understanding of genomic sequences obtained from the pre-trained foundation model with the task-specific dataset for virus identification. First, ViraLM is built with a 12-layer attention block, followed by a fully connected layer that serves as a binary classifier for classifying input sequences as viruses or nonviruses. The learnable parameters of the attention block in ViraLM are initialized by transferring values from the corresponding parameters in DNABERT-2’s attention block, ensuring that the model starts with the parameters and representations learned by DNABERT-2. Then, unlike DNABERT-2’s self-supervised training, ViraLM is trained using labeled data, which consists of positive samples (virus sequences) and negative samples (nonvirus sequences) generated from high-quality genomes. During fine-tuning, the learnable parameters are aligned with the specific training objective of virus identification. To facilitate parameter updates, binary cross-entropy loss and the Adam optimizer with a learning rate of 1e−5 and a warm-up step of 50 are used. This approach leverages the strengths of both self-supervised and supervised learning, enabling the model to gain a comprehensive understanding of genomic patterns and contexts while being specifically tailored to virus identification. Once the fine-tuning process is completed, the model becomes capable of accurately identifying virus sequences from the input data.

### 2.2 Construction of training dataset

To construct a task-specific training dataset, we integrated two virus datasets from different sources to generate positive samples. First, we downloaded the complete viral genomes released before September 2023 from the National Center for Biotechnology Information (NCBI) RefSeq database (https://www.ncbi.nlm.nih.gov/). The RefSeq viral genomes were divided based on their realm-rank taxons: *Adnaviria*, *Duplodnaviria*, *Monodnaviria*, *Riboviria*, *Ribozyviria*, and *Varidnaviria*. Specifically, we combined *Adnaviria*, *Ribozyviria*, and genomes that lack realm-rank taxons into one group due to their small sizes. Second, we downloaded a dataset of high-quality viral genomes used in VirSorter2 (Guo *et al.* 2021), which were carefully collected and curated in the previous works. These genomes represent a set of novel viruses that are dissimilar to RefSeq reference genomes. They were pre-grouped into dsDNA phages, *Lavidaviridae* (virophages), NCLDVs (giant viruses), and RNA viruses. In total, the virus dataset contains 49 929 genomes.

When considering negative samples for training, it is impractical to include all possible nonviral genomes. Instead, a rigorous sampling approach was used, focusing on sequences that are prone to being misclassified as viruses. These include the host genomes of prokaryotic viruses, such as bacteria and archaea, as well as eukaryotes such as insects, which serve as hosts or key vectors for viruses (Guo *et al.* 2021, Shang and Sun 2022, Rozo-Lopez *et al.* 2023). By incorporating these challenging cases, the model can learn a more effective decision boundary between viruses and nonviral entities. First, we downloaded complete assemblies of prokaryotes (bacteria and archaea) and eukaryotes (fungi and protozoa) from the NCBI RefSeq database. These carefully selected genomes represent typical nonviral entities commonly appearing in metagenomic data (Doytchinov and Dimov 2022, Wang *et al.* 2022). In addition, we also include the plasmid data from the RefSeq database, which is prone to be misidentified by the existing virus identification tools. Furthermore, considering that insects serve as hosts and key vectors for carrying a wide range of viruses, including arthropod-borne viruses (arboviruses) that pose significant public health concerns (Girard *et al.* 2020), we also include the chromosomes from the *Insecta*. Due to the significantly larger size of the bacteria and *Insecta* datasets compared to the other nonviral organisms, we down-sampled two genomes per genus from the bacteria datasets and one genome per genus from the *Insecta* dataset. To remove the possible endogenous virus elements in nonviral sequences that might confuse the model, we applied BLASTN (Altschul *et al.* 1990) to align the nonviral sequences against the viral sequences and removed the common regions from the sequences.

Finally, the genomes within each group (positive and negative) were randomly divided into training and test sets with a ratio of nine to one. This separation was performed individually for each realm-rank taxon to ensure a balanced distribution of different organisms in the datasets. The genomes were randomly cut into short contigs ranging from 300 to 2 kb following the uniform distribution to simulate variable-length contigs in the metagenomic data. To mitigate data imbalance caused by significant differences in genome sizes, the fragments from the nonviral genomes were down-sampled to match the size of the virus dataset. As a result, we constructed an extensive training set consisting of 40 billion nucleotide bases from bacteria, archaea, plasmid, fungi, protozoa, *Insecta*, and viruses. The large volume and diverse composition of the training data enable the model to gather ample information, enhancing its ability to generalize effectively when processing previously unseen species. We rigorously tested ViraLM on multiple datasets with increasing complexity. Detailed information of the test data can be found in Supplementary Section S1.2.

## 3 Result

We compare ViraLM against new or state-of-the-art methods including geNomad (Camargo *et al.* 2023), VIRify (Rangel-Pineros *et al.* 2023), VirSorter2 (Guo *et al.* 2021), VIBRANT (Kieft *et al.* 2020), VirRep (Dong *et al.* 2024), and DeepVirFinder (Ren *et al.* 2020). The settings for running each tool are detailed in Supplementary Section S2.

### 3.1 ViraLM outperforms the benchmarked methods

The first test set consists of virus sequences from NCBI RefSeq and uncultured viral genomes obtained from metagenomes as detailed in Supplementary Section S1.2. In order to evaluate whether prokaryotic or eukaryotic sequences pose a bigger challenge for virus detection, we evaluate and report the performance on the two types of negative test data separately.

#### 3.1.1 Prokaryotic genomes

First, we report the performance on the negative test set containing sequences from bacteria, archaea, and plasmids. To ensure a balanced test set, we choose an equal number of negative sequences as the viral sequences. The area under the ROC curve (Supplementary Fig. S4) reveals that ViraLM returns more reliable results under all classification thresholds compared with other tools. We further analyzed the virus identification performance of different tools on contigs of different length ranges. In general, the shorter the contig, the harder to distinguish viruses from others. The results can be found in Fig. 1B. ViraLM outperforms other methods across all length ranges, highlighting its robustness for virus identification. Specifically, VIBRANT and VirRep cannot predict on contigs shorter than 1 kb and therefore their scores are zero. Protein-based methods (VirSorter2, VIBRANT, and geNomad) perform well on contigs over 10 kb but struggle with short contigs due to their reliance on complete protein information, which is less likely to be present in shorter sequences. On the contrary, ViraLM, VirRep, and DeepVirFinder rank highest on contigs between 1 and 2 kb, highlighting the strength of nucleotide-based methods. We also investigate ViraLM’s performance under various thresholds from 0.5 to 0.9. The results (Supplementary Fig. S10) suggest that ViraLM achieves its highest F1-score at the default threshold of 0.5, representing the best trade-off between precision and recall. Alternatively, users may choose a stricter threshold to increase precision at the cost of recall, depending on their specific requirements.

#### 3.1.2 Eukaryotic genomes

Next, we follow the design in (Guo *et al.* 2021) and extend our assessment to eukaryotic genomes, broadening the scope beyond small eukaryotes like fungi and protozoa. We conduct a comparative analysis of all methods on contigs of mixed length where negative test samples only contain contigs from five distinct eukaryotic groups (fungi, protozoa, insects, bats, and humans), which are frequently found in host-associated microbiomes. The initial observation indicates that the ability of all tools except VirRep and VIRify to differentiate viral sequences from those of prokaryotic or eukaryotic origin is remarkably consistent, as evidenced by Fig. 1C. A more detailed comparison of the tools’ precision, recall, and F1-score for each eukaryotic group is presented in Fig. 1D.

Upon assessing the classification results, we find that ViraLM delivers an enhanced overall performance compared to other methods. In general, ViraLM, VirSoter2, VIBRANT, and geNomad demonstrate high precision in all eukaryotic groups. The well-constructed viral hallmark gene databases contribute to the high precision of protein-based methods. However, VirSoter2, VIBRANT, and geNomad exhibit low recall, indicating that they are conservative models that are prone to generate fewer false positives but will miss a considerable number of true positives. In contrast, ViraLM achieves comparable precision to that of the protein-based tools while maintaining high recall across all groups. In particular, its F1-scores on distinguishing viruses from bat and human-originated contigs are 0.91 and 0.92, respectively. As the closest species in the labeled training data for ViraLM is merely *Insecta*, achieving such high F1-scores underscores ViraLM’s adaptability and efficacy in analyzing metagenomic data derived from various and new host organisms.

### 3.2 Performance on IMG/VR dataset

We chose four methods (ViraLM, VirRep, VirSorter2, and geNomad) that performed the best in the previous experiments and benchmarked them on the IMG/VR dataset (Supplementary Section S1.2) consisting of 908 726 viral contigs that are cut into fixed-length (2 and 5 kb) with various numbers (zero to five) of complete proteins. The results (Fig. 1E) reveal that ViraLM and VirRep identify most of the viral contigs across different protein densities. In particular, both tools accurately predict the contigs from noncoding regions, whereas protein-based tools commonly fail, demonstrating the strength of nucleotide-based tools on short contigs. While VirRep predicts the most viruses, previous results (Fig. 1D) indicate that it tends to produce false positives. Overall, the comparison across various contig lengths and protein densities highlights ViraLM as a reliable tool for uncovering the extensive diversity of viruses.

Finally, we run the methods on three public microbiome datasets released in previous studies. ViraLM presents the best and most robust performance across different environmental samples (Supplementary Fig. S14). We also considered the possibility of chimeric contigs in metagenomic data. The results in Supplementary Fig. S15 demonstrate that ViraLM tends to retain pure viral contigs and discard highly contaminated contigs. These experiments are detailed in Supplementary Sections S2.3 and S2.4.

## 4 Discussion

In this work, we present a novel language model, named ViraLM, for virus identification. The major improvement of our method stems from the adoption of the pre-trained foundation model and our careful construction of the extensive training set. The pre-trained foundation model provides extensive knowledge of the intricate patterns and relationships within genomes. Built on this prior knowledge, our model is able to recognize the subtle differences hidden in the nucleotide sequences between viruses and other organisms, leading to improved performance. In addition, we construct a vast dataset with novel uncultured viruses and distinct groups of nonvirus organisms. This inclusion of additional genomes expands the diversity of our training data, enhancing the model’s generalization capability. Through benchmark experiments conducted on standard benchmark datasets, IMG/VR datasets, and real metagenomic data, our model consistently outperforms existing methods across various scenarios. In particular, it achieves a remarkable 22% improvement in F1-score on short contigs. Moreover, our model exhibits superior robustness when confronted with complex real metagenomic samples, highlighting its adaptability and advanced performance in different environments.

Although ViraLM has greatly improved virus identification, there are still some limitations. First, ViraLM is designed to be a lightweight and easy-to-use tool that does not rely on references. As a result, unlike VIBRANT and geNomad, ViraLM does not provide users with taxonomy or gene annotation information for the identified viral contigs. Second, although language models have the ability to capture long-range interactions in nucleotide sequences, they often encounter significant computational challenges when dealing with lengthy input sequences. This is particularly relevant considering that nucleotide sequences can reach lengths of millions of base pairs (bp). Thus, we have several goals to optimize or extend ViraLM in our future work. First, we will explore the incorporation of additional domain knowledge into our framework to enhance the performance of ViraLM in identifying viral fragments. Second, we will actively keep pace with the advancements in deep learning to maintain the computational efficiency of ViraLM and overcome the length limitation.

## Supplementary Material

btae704_Supplementary_Data

## Data Availability

All data and codes used for this study are available online via: https://github.com/ChengPENG-wolf/ViraLM.

## References

[btae704-B1] Altschul SF , Gish W, Miller W et al Basic local alignment search tool. J Mol Biol 1990;215:403–10. 2231712 10.1016/S0022-2836(05)80360-2

[btae704-B2] Camargo AP , Roux S, Schulz F et al Identification of mobile genetic elements with genomad. Nat Biotechnol 2023;42:1–10. 10.1038/s41587-023-01953-y PMC11324519 37735266

[btae704-B3] Dalla-Torre H , Gonzalez L, Mendoza-Revilla J et al The nucleotide transformer: building and evaluating robust foundation models for human genomics. bioRxiv, 2023, preprint: not peer reviewed. 10.1038/s41592-024-02523-z PMC11810778 39609566

[btae704-B4] Devlin J , Chang M-W, Lee K et al Bert: pre-training of deep bidirectional transformers for language understanding. arXiv, arXiv:1810.04805, 2018, preprint: not peer reviewed.

[btae704-B5] Dong Y , Chen W-H, Zhao X-M. Virrep: a hybrid language representation learning framework for identifying viruses from human gut metagenomes. Genome Biol 2024;25:177. 38965579 10.1186/s13059-024-03320-9 PMC11229495

[btae704-B6] Doytchinov VV , Dimov SG. Microbial community composition of the antarctic ecosystems: review of the bacteria, fungi, and archaea identified through an NGS-based metagenomics approach. Life 2022;12:916. 35743947 10.3390/life12060916 PMC9228076

[btae704-B7] Girard M , Nelson CB, Picot V et al Arboviruses: a global public health threat. Vaccine 2020;38:3989–94. 32336601 10.1016/j.vaccine.2020.04.011 PMC7180381

[btae704-B8] Guo J , Bolduc B, Zayed AA et al Virsorter2: a multi-classifier, expert-guided approach to detect diverse dna and rna viruses. Microbiome 2021;9:37. 33522966 10.1186/s40168-020-00990-y PMC7852108

[btae704-B9] Kieft K , Zhou Z, Anantharaman K. Vibrant: automated recovery, annotation and curation of microbial viruses, and evaluation of viral community function from genomic sequences. Microbiome 2020;8:90. 32522236 10.1186/s40168-020-00867-0 PMC7288430

[btae704-B10] Koonin EV , Krupovic M, Dolja VV. The global virome: How much diversity and how many independent origins? Environ Microbiol 2023;25:40–4. 36097140 10.1111/1462-2920.16207

[btae704-B11] Rangel-Pineros G , Almeida A, Beracochea M et al Virify: an integrated detection, annotation and taxonomic classification pipeline using virus-specific protein profile hidden Markov models. PLoS Comput Biol 2023;19:e1011422. 37639475 10.1371/journal.pcbi.1011422 PMC10491390

[btae704-B12] Ren J , Song K, Deng C et al Identifying viruses from metagenomic data using deep learning. Quant Biol 2020;8:64–77. 34084563 10.1007/s40484-019-0187-4 PMC8172088

[btae704-B13] Rozo-Lopez P , Brewer W, Käfer S et al Untangling an insect’s virome from its endogenous viral elements. BMC Genomics 2023;24:636. 37875824 10.1186/s12864-023-09737-z PMC10594914

[btae704-B14] Shang J , Sun Y. Cherry: a computational method for accurate prediction of virus–prokaryotic interactions using a graph encoder–decoder model. Brief Bioinform 2022;23:bbac182. 35595715 10.1093/bib/bbac182 PMC9487644

[btae704-B15] Wang J , Gou Q-y, Luo G-y et al Total rna sequencing of phlebotomus chinensis sandflies in China revealed viral, bacterial, and eukaryotic microbes potentially pathogenic to humans. Emerg Microbes Infect 2022;11:2080–92. 35916448 10.1080/22221751.2022.2109516 PMC9448391

[btae704-B16] Zhou Z , Ji Y, Li W et al Dnabert-2: efficient foundation model and benchmark for multi-species genome. arXiv, arXiv:2306.15006, 2023, preprint: not peer reviewed.

