# Supplementary material for: ViraLM: empowering virus discovery through the genome foundation model
Source: Bioinformatics. 2024 Nov 23;40(12):btae704. doi: 10.1093/bioinformatics/btae704 (PMC11631183; doi:10.1093/bioinformatics/btae704)
Supplement: btae704_Supplementary_Data [file btae704_supplementary_data.pdf]

1 Supplementary for Methods and materials

1.1 The architecture of ViraLM

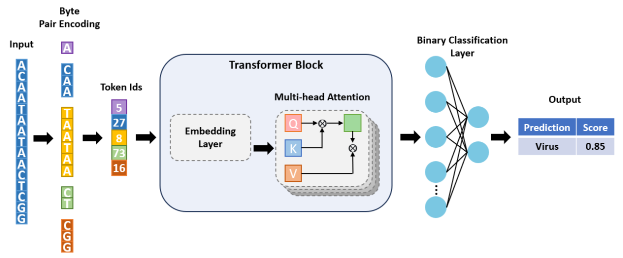

Fig. S1. The framework of ViraLM. The main architecture of ViraLM contains three parts: a Byte-Pair Encoding block, a multi-layer Transformer block, and a specific binary classifier layer for virus classification.

1.1.1 Byte-Pair Encoding

In natural language processing, sentences can be divided into words based on semantics and grammar. However, genomic sequences lack such inherent “words”, posing a challenge for effective tokenization. Previous methods like fixed-length k-mer tokenization (Dong *et al.*, 2024; Dalla-Torre *et al.*, 2023) face information leakage issues due to overlapping regions between adjacent k-mers, while non-overlapping k-mers are sensitive to single nucleotide mutations (Ji *et al.*, 2021).

To address these issues, Byte-Pair Encoding (BPE) is employed (Sennrich *et al.*, 2015). BPE is a statistical subword algorithm that learns a vocabulary of variable-length tokens from frequent nucleotide pairs. BPE starts with individual nucleotides (A, C, G, and T) as tokens, then merges frequent pairs iteratively, forming a vocabulary that captures biologically significant subwords.

With the vocabulary generated by BPE, the input nucleotide sequences are translated into a sequence of tokens. Specifically, sequences containing ambiguous bases (letters other than ACGT) will be discarded. An illustration of this process is provided in Fig. S1, where an input sequence is divided into five tokens of varying lengths: A, CAA, TAATAA, CT, and CGG. Then each token in the sequence is assigned a unique identifier known as a token ID. These sequences of token IDs serve as the input for the transformer block, allowing the model to effectively learn and capture the relationships and patterns within the genomic data.

1.1.2 Transformer block

Because even short nucleotide sequences of 1kbp length can contain more than 230 BPE tokens, convolution and recurrence in CNNs and RNNs often struggle to capture these long-term token dependencies due to their fixed window size or vanishing gradients issue. To address this challenge, ViraLM employs a 12-layer Transformer (Vaswani *et al.*, 2017) to obtain contextual information within the sequences. In the transformer block, the tokenized sequence is first encoded into a matrix representation, denoted as $M \in \mathbb{R}^{\mathrm{L}\times\mathrm{embed}}$. Here, each token is mapped to a high-dimensional embedding space, where L represents the length of the tokenized sequence, and embed corresponds to the dimensionality of the embedding space. This encoding process ensures that tokens with semantic relationships are positioned closely together in the high-dimensional embedding space, enabling effective representation of the contextual and semantic information between different tokens. Then, the matrix is fed into the multi-head self-attention layer that can be expressed as Eqn. 1.

$$\begin{cases} \mathrm{MultiHead}(M) = \mathrm{Concat}\left(\mathrm{head}_1, \ldots, \mathrm{head}_h\right) W^0 \\ \mathrm{head}_i = \mathrm{softmax}\left(\frac{Q_i \cdot K_i^T}{\sqrt{d_k}}\right) V_i \\ Q_i = M \cdot W_i^Q \\ K_i = M \cdot W_i^K \\ V_i = M \cdot W_i^V \end{cases} \tag{1}$$

Where the input matrix M is projected into queries (Q), keys (K), and values (V) in dimension d_k for each attention head $head_i$ separately. W^0, W_i^Q, W_i^K and W_i^V ($i = 1, 2, ..., h$) are learned matrices of the linear projection. Here, the query represents the information being sought, while the key serves as a reference for comparison. By computing attention scores between query-key pairs, the model determines the relationship between tokens. These attention scores are then used to weigh the corresponding values. By projecting the embedded matrix into separate queries, keys, and values for each attention head, the model can capture diverse perspectives and learn different representations of the sequence. This multi-head attention mechanism allows access to all features in the sequence regardless of token distance or order, enabling the model to assign distinct attention weights to individual tokens based on their relevance to one another, effectively leveraging the relationships between the sequence motifs in the sequence.

1.2 Test datasets

Labeled data for test As described in section 2.2 in the main paper, the genomes are randomly divided into training and test sets with a ratio of nine to one. To ensure a fair evaluation of the model, we examine the sequences in the test set to exclude those that exhibit high similarity with the training sequences. This is achieved by aligning the test sequences using BLASTN to the training data. Alignments from different regions of the same query sequence are aggregated to calculate the overall alignment coverage and identity. According to the guidelines provided by the International Committee on Taxonomy of Viruses (ICTV) (Moraru *et al.*, 2020), a similarity threshold of 70% is established for classification at the genus level. To ensure a rigorous testing process, we adopt a more stringent threshold, requiring an overall coverage of less than 50% between training and test sequences. Thus, the test set can provide a fair and unbiased assessment of the model’s performance. In addition, we include in our test set the reference bat genome (order *Chiroptera*) and human genome to further test the model’s generalization ability on other large eukaryotic organisms that are not included in our training data. Similar to previous steps, these sequences are also fragmented into short contigs (500bp to 20kbp). In particular, all the test sequences are sampled to balance the positive samples and negative samples, ensuring a fair evaluation.

IMG/VR dataset The Integrated Microbial Genome/Virus (IMG/VR) v4 database (Camargo *et al.*, 2023b) contains 5,576,197 high-confidence viral genomes collected from different environment samples. These genomes are systematically quality-checked and classified into high-quality, medium-quality, low-quality, and unsure-quality genomes by IMG/VR. We recruit 495,576 high-quality genomes from the database. These genomes are aligned against the training set to remove highly similar sequences with BLASTN (i.e., overall coverage >50%). After the removal process, we get a total of 367,782 genomes, which represent a broad range of novel uncultured viruses. These diverse viruses may exhibit various protein densities. To test how the number of proteins on a contig can affect the performance of virus identification, we first predict the open reading frames using Prodigal (Hyatt *et al.*, 2010). Then the genomes are cut into 2kbp length fragments with 0 (non-coding regions), 1 and 2 proteins, and 5kbp length fragments with 3, 4, and 5 proteins, respectively.

Microbiome sequencing dataset We retrieved two public real metagenomic data and one RNA-Seq data sampled from different environments, including water (Wang *et al.*, 2024), soil (Wang *et al.*, 2021), and insect-associated ecosystems (Wang *et al.*, 2022), respectively. The water metagenome is sequenced from a total of 30 surficial water samples collected from Jinghu Lake (China), a lake that is replenished by reclaimed water. The soil microbiome comes from *Phragmites* plants rhizosphere soil (soil that is firmly attached to root) and bulk soil (nearby soil without *Phragmites* plants). The insect microbiome is obtained from hundreds of sandflies. Low-quality reads and adaptor sequences are removed from the raw sequencing data using Fastp (Chen, 2023). Then the resulting clean reads are assembled into contigs using Megahit (Li *et al.*, 2015). Only the contigs with lengths over 2kbp are reserved for prediction, resulting in 1,294,985, 461,310, and 149,322 contigs in the three types of samples, respectively.

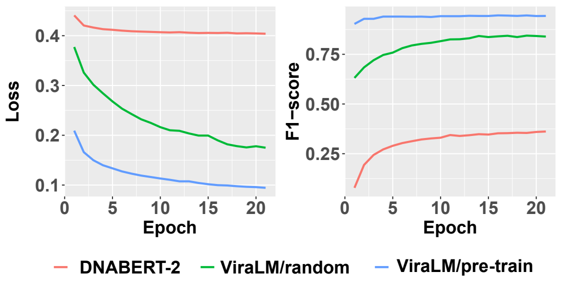

Fig. S2. Comparison of training loss and F1-score between the model initialized using the pre-trained foundation model, the model with randomly initialized weights, and DNABERT-2 alone. The model initialized using the pre-trained foundation model converges faster and performs better.

2 Supplementary for Result

We evaluate ViraLM on various test datasets and compare it against new or state-of-the-art methods including VirRep (Dong *et al.*, 2024), geNomad (Camargo *et al.*, 2023a), VIRify (Rangel-Pineros *et al.*, 2023), VirSorter2 (Guo *et al.*, 2021), VIBRANT (Kieft *et al.*, 2020) and DeepVirFinder (Ren *et al.*, 2020). Because VirRep, geNomad, VIRify, VirSorter2, and VIBRANT either do not support retraining or rely on their curated category-specific protein databases for prediction, we directly run these tools without retraining. Although DeepVirFinder supports retraining, the provided script failed to process a medium-size dataset during the encoding step due to the large memory usage. Specifically, a 300 MB input FASTA file requires approximately 14 GB of memory to store the encoded sequences. This high memory demand poses a significant barrier to retraining with the extensive datasets (over 40 GB) used for ViraLM. We run VirSorter2 (v2.2.4) with the option “–include-groups dsDNAphage,NCLDV,RNA,ssDNA,lavidaviridae” to predict viruses from all the groups. VIBRANT (v1.2.1), geNomad (v1.7.4), DeepVirFinder (v1.0), VIRify (v2.0.1), and VirRep are run on their default settings. Similarly, during all the following tests, ViraLM is run under its default setting without any extra adjustment for different test datasets.

2.1 Ablation study in ViraLM

As ViraLM is built upon a pre-trained foundation model, we first investigate whether ViraLM’s training process, which adjusts the DNABERT-2’s parameters based on our virus identification task, brings any benefits, compared with starting from random parameters in the Transformer block. Specifically, we compare the performance of ViraLM using two different weight initialization strategies: random initialization and initialization with the pre-trained foundation model. For the randomly-initialized setting, we construct a new model with the same structure as ViraLM. In this model, all the learnable weights in the transformer block

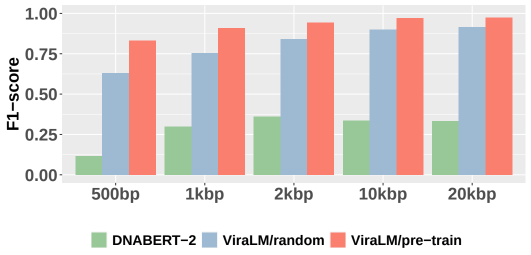

Fig. S3. Comparison of virus identification performance on contigs of various lengths between the model initialized using the pre-trained foundation model, the model with randomly initialized weights, and DNABERT-2 alone.

are randomly initialized using a normal distribution with a mean of zero and a standard deviation of 0.02. The biases in the model are initialized to zero.

Table S1. The F1-score of ViraLM trained with fixed warm-up steps and different learning rates.

Learning rate \ Epoch	1	2	3	4	5
1e-4	0	0	0	0	0
1e-5	0.48	**0.64**	**0.70**	**0.71**	**0.73**
1e-6	**0.52**	0.58	0.56	0.57	0.55

A hyperparameter search was conducted to find the optimal hyperparameters for the model. We trained the model with various combinations of learning rates and warm-up steps on a small subset of the training data and evaluated their performances on the test data. First, we set the warm-up steps as 100 and studied the effect of learning rates of $1e-4$, $1e-5$, and $1e-6$. The performances measured in F1-score are shown in Table S1. A small learning rate ($1e-6$), impedes the model’s ability to learn from the training data, while a large learning rate ($1e-4$) leads to the exploding gradient issue.

Table S2. The F1-score of ViraLM trained with fixed learning rate and different warm-up steps.

Warm-up steps \ Epoch	1	2	3	4	5
50	**0.62**	**0.69**	0.67	**0.72**	**0.73**
100	0.48	0.64	**0.70**	0.71	0.73
200	0.43	0.64	0.67	0.72	0.73

Because $1e-5$ is the best learning rate as shown in Table S1, we fixed the learning rate as $1e-5$ and adjusted the warm-up steps from 50 to 200 to show how the number of warm-up steps can affect the performance. The findings, detailed in Table S2, reveal that shorter warm-up steps (50) facilitate a faster training process.

Consequently, for the final model configuration, we employ a learning rate of 1e-5 coupled with a warm-up step of 50 to train the model. During the training process, we keep track of the training losses and F1-score for both models on the test set in each epoch. As shown in Fig. S2, the pre-trained model demonstrates faster convergence compared to the model with random initialization, resulting in a superior performance with the same number of epochs. The observed trends highlight the importance of the foundation model as a valuable starting point for improving model accuracy and speeding up the training process. We also compare their final performance on contigs with various lengths in

Fig. S3. The results show that the pre-trained model outperforms the model with random initialization across all contig ranges, underscoring the effectiveness of the foundational model in boosting overall performance. In addition, we evaluate the virus identification capacity of DNABERT-2 alone, without further adjusting its weights on the virus identification task. We use DNABERT-2 to generate embedding and then feed it into the same classifier used in ViraLM. During training, only the classifier’s parameters are updated. The results in Fig. S2 and Fig. S3 indicate that while the foundational model provides a valuable starting point for model initialization, DNABERT-2 alone cannot be directly used for downstream tasks. This is mainly because DNABERT-2 was not pre-trained on viruses, resulting in poor embeddings of viral genomes. These findings suggest that the virus identification task is complex and that the model requires further training to effectively capture the extensive information inherent in the diverse virus community.

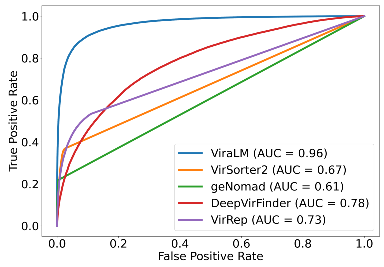

Fig. S4. ROC curves on mixed length contigs where negative samples only consist of prokaryotes (bacteria, archaea, plasmid). The value in the parentheses represents the AUC for each tool.

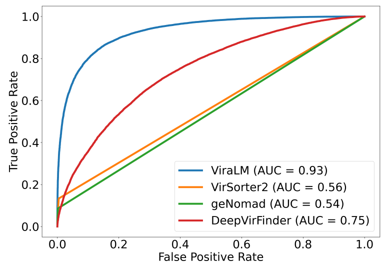

Fig. S5. ROC curves on contigs of length 500bp.

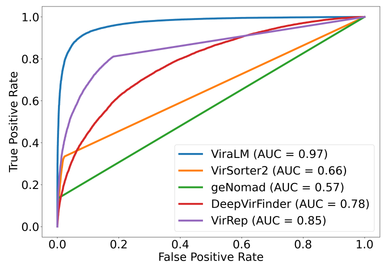

Fig. S6. ROC curves on contigs of length 1kbp.

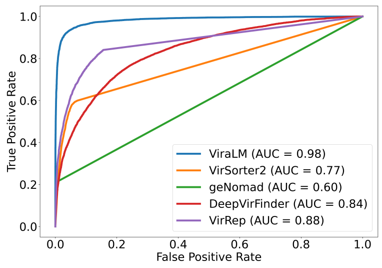

Fig. S7. ROC curves on contigs of length 2kbp.

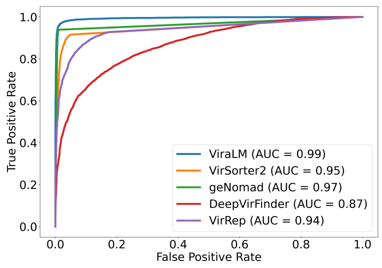

Fig. S8. ROC curves on contigs of length 10kbp.

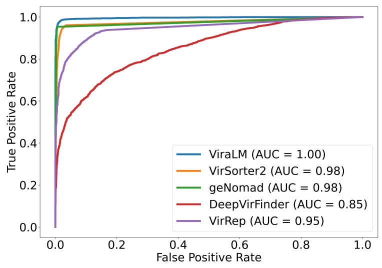

Fig. S9. ROC curves on contigs of length 20kbp.

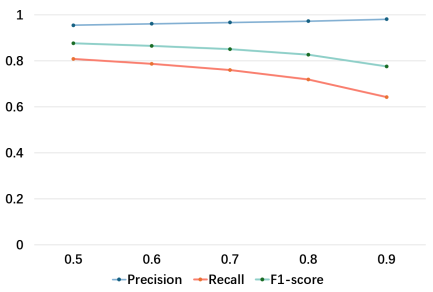

Fig. S10. ViraLM’s performance under various thresholds from 0.5 to 0.9. ViraLM achieves its highest F1-score at the default threshold of 0.5, representing the best trade-off between precision and recall. Alternatively, users may choose a stricter threshold to increase precision at the cost of recall, depending on their specific requirements.

2.2 Benchmark on the test set

We report the performance on the negative test set containing sequences from bacteria, archaea, and plasmids. Still, to ensure a balanced test set, we choose an equal number of negative sequences as the viral sequences. After obtaining the prediction results, we draw a ROC curve using the prediction scores of each tool in Fig. S4. While Fig. S4 is generated on all the contigs in the test set, we further analyzed the virus identification performance of different tools on contigs of different length ranges (Fig. S5, S6, S7, S8, and S9). Specifically, because VIBRANT and VIRify do not provide a score associated with each prediction, they are not plotted in the figures. The area under the ROC curve reveals that ViraLM returns more reliable results under all classification thresholds compared with other tools.

2.3 Performance on microbiome sequencing data

we run ViraLM, VirRep, VirSorter2, and geNomad on two public real metagenomic datasets and one RNA-Seq dataset released in previous studies (Wang *et al.*, 2024, 2021, 2022). These datasets, derived from distinct ecosystems such as aquatic environments, terrestrial soils, and insect populations, represent a wide range of microbial communities. In total, there are 1,294,985, 461,310, and 149,322 contigs with lengths over 2kbp, respectively. The number of viruses predicted by each tool and the overlap (intersection) among different groups is displayed by UpSet Plots in Fig. S11, S12, and S13. Across all the experiments, VirRep always returns the largest number of predicted viral contigs. As shown in Fig. 1 D in the main manuscript, VirRep has lower precision than other tools, suggesting that some of its returned predictions are not likely viruses. Fig. S11 and S13 exhibit a unanimous identification of 131,585 viruses in the water sample made by all tools, whereas in the insect microbiome, the tools' predictions have small overlaps. This observation reveals that different ecosystems have different virus composition and complexity.

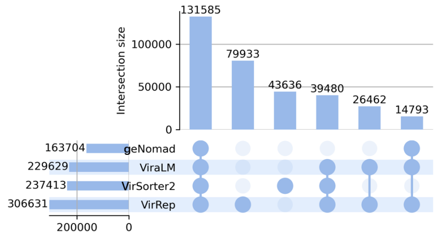

Fig. S11. Comparison of the overlap of predicted viruses from surficial water samples. Horizontal bars represent the number of viruses predicted by each tool. Each column represents a set of viruses. The vertical bar on top of the column shows the size of the set. The filled-in cells joined by a line indicate which groups share these viruses. The bars are sorted by the size of the intersection.

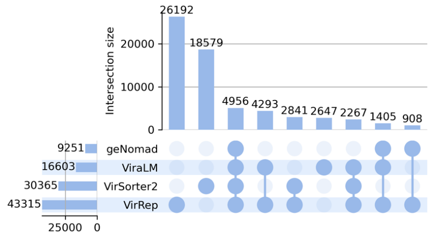

Fig. S12. Comparison of the overlap of predicted viruses from plants rhizosphere soil samples.

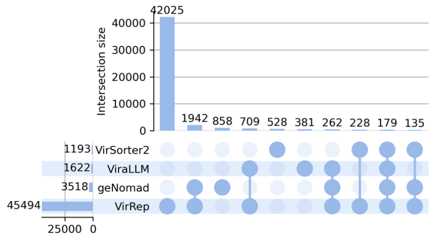

Fig. S13. Comparison of the overlap of predicted viruses from sandfly samples.

2.4 Implication of chimeric contigs to virus identification

We investigate the impact of chimeric contigs (sequences that combine segments from viral and bacterial origins) on virus identification. We randomly pair virus fragments with bacteria fragments to simulate chimeric contigs with increasing contamination levels (0%, 10%, 20%, and 50%). Fig S15 shows the performance of ViraLM on 19,271 simulated chimeric contigs. The results demonstrate that ViraLM and geNomad tend to retain only viral contigs when contamination is low. As the contamination level increases to around 50%, ViraLM will discard them and will not report them as viruses anymore. On the contrary, VirSorter2 and VirRep can detect virus-host boundaries and extract proviruses that are integrated into host genomes.

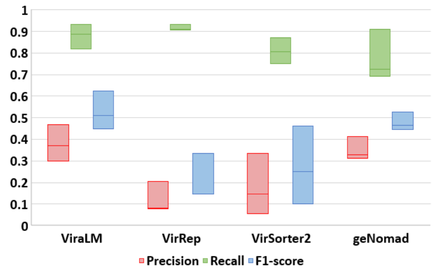

Fig. S14. The performance on three microbiome sequencing data by each tool. X-axis: the names of the tools. Y-axis: the value of the metrics on three different samples.

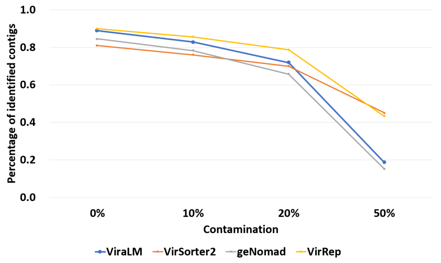

Fig. S15. Implication of chimeric contigs to virus identification.

Although we lack the ground truth about the viral composition in these real data, we can decide the labels of some contigs based on very strict alignment results against reference genomes. We categorize the contigs into bacteria, archaea, eukaryotes, and viruses by assigning labels according to the best-hit BLASTN alignment against the NT database with a strict threshold (identity >85% and coverage >85%). As a result, 21,168, 31,119, and 1,292 contigs are classified into one of these groups. By summarizing the BLASTN results and analyzing the composition of the samples, we found that non-virus contigs outnumber virus contigs by approximately 28 to 316 times. Such pronounced imbalance can inherently impact the precision calculations across all methods. The performance of all the tools on these labeled subsets is shown in Fig. S14. The result confirms that VirRep tends to overestimate the virus population, resulting in low precision. VirSorter2 exhibits wide ranges of precision, varying from 0.05 to 0.33, demonstrating its limitation on specific environmental samples. In general, ViraLM presents the highest and relatively stable performance in microbiome sequencing data sampled from different environments. As mentioned earlier, as the number of non-viral contigs is significantly larger than viral contigs, precision is naturally lower even when the false positive (FP) rate is low. For example, the FP rate of ViraLM in the water sample is 0.011, indicating that only 1% of the non-viral contigs are misclassified as viruses. However, given the large

Program	ViraLM	VirSorter2	DeepVirFinder	VIBRANT	geNomad	VirRep	VIRify
Elapsed time(min)	59 (CPU) / 3 (GPU)	156	11	38	21	4 (GPU)	454
Disk size(MB)	447	12288	114	11264	1433	54	28160

Table S3. The elapsed time to make predictions on the test set and the size of disk space needed by the tools.

total number of non-viral contigs, a low FP rate can still contribute to a low precision calculation.

2.5 Time efficiency and disk size

We run ViraLM, VirSorter2, DeepVirFinder, VIBRANT, VIRify, and geNomad on the Intel® Xeon® Gold 6258R CPU with 8 cores. Considering that ViraLM and VirRep are large deep-learning models and the relative inefficiency of CPUs in handling floating-point arithmetic, we run ViraLM and VirRep on a single NVIDIA GeForce RTX 3090 GPU. All the methods are run on a test set containing 5,000 contigs of length 10kbp. The elapsed time is shown in Table S3. Additionally, we record the sizes of disk space needed by the tools, predominantly comprising the databases and models. The rapid prediction capability and relatively compact size suggest that ViraLM is a lightweight and efficient tool that may assist and accelerate the discovery of new viruses.

References

Camargo, A. P., Roux, S., Schulz, F., Babinski, M., Xu, Y., Hu, B., Chain, P. S., Nayfach, S., and Kyrpides, N. C. (2023a). Identification of mobile genetic elements with genomad. *Nature Biotechnology*, pages 1–10.

Camargo, A. P., Nayfach, S., Chen, I.-M. A., Palaniappan, K., Ratner, A., Chu, K., Ritter, S. J., Reddy, T., Mukherjee, S., Schulz, F., *et al.* (2023b). Img/vr v4: an expanded database of uncultivated virus genomes within a framework of extensive functional, taxonomic, and ecological metadata. *Nucleic acids research*, **51**(D1), D733–D743.

Chen, S. (2023). Ultrafast one-pass fastq data preprocessing, quality control, and deduplication using fastp. *iMeta*, page e107.

Dalla-Torre, H., Gonzalez, L., Mendoza-Revilla, J., Carranza, N. L., Grzywaczewski, A. H., Oteri, F., Dallago, C., Trop, E., de Almeida, B. P., Sirelkhatim, H., *et al.* (2023). The nucleotide transformer: Building and evaluating robust foundation models for human genomics. *bioRxiv*, pages 2023–01.

Dong, Y., Chen, W.-H., and Zhao, X.-M. (2024). Virrep: a hybrid language representation learning framework for identifying viruses from human gut metagenomes. *Genome Biology*, **25**(1), 177.

Guo, J., Bolduc, B., Zayed, A. A., Varsani, A., Dominguez-Huerta, G., Delmont, T. O., Pratama, A. A., Gazitúa, M. C., Vik, D., Sullivan, M. B., *et al.* (2021). Virsorter2: a multi-classifier, expert-guided approach to detect diverse dna and rna viruses. *Microbiome*, **9**, 1–13.

Hyatt, D., Chen, G.-L., LoCascio, P. F., Land, M. L., Larimer, F. W., and Hauser, L. J. (2010). Prodigal: prokaryotic gene recognition and translation initiation site identification. *BMC bioinformatics*, **11**, 1–11.

Ji, Y., Zhou, Z., Liu, H., and Davuluri, R. V. (2021). Dnabert: pre-trained bidirectional encoder representations from transformers model for dna-language in genome. *Bioinformatics*, **37**(15), 2112–2120.

Kieft, K., Zhou, Z., and Anantharaman, K. (2020). Vibrant: automated recovery, annotation and curation of microbial viruses, and evaluation of viral community function from genomic sequences. *Microbiome*, **8**(1), 1–23.

Li, D., Liu, C.-M., Luo, R., Sadakane, K., and Lam, T.-W. (2015). Megahit: an ultra-fast single-node solution for large and complex metagenomics assembly via succinct de bruijn graph. *Bioinformatics*, **31**(10), 1674–1676.

Moraru, C., Varsani, A., and Kropinski, A. M. (2020). Viridic—a novel tool to calculate the intergenomic similarities of prokaryote-infecting viruses. *Viruses*, **12**(11), 1268.

Rangel-Pineros, G., Almeida, A., Beracochea, M., Sakharova, E., Marz, M., Reyes Muñoz, A., Hölzer, M., and Finn, R. D. (2023). Virify: An integrated detection, annotation and taxonomic classification pipeline using virus-specific protein profile hidden markov models. *PLOS Computational Biology*, **19**(8), e1011422.

Ren, J., Song, K., Deng, C., Ahlgren, N. A., Fuhrman, J. A., Li, Y., Xie, X., Poplin, R., and Sun, F. (2020). Identifying viruses from metagenomic data using deep learning. *Quantitative Biology*, **8**, 64–77.

Sennrich, R., Haddow, B., and Birch, A. (2015). Neural machine translation of rare words with subword units. *arXiv preprint arXiv:1508.07909*.

Vaswani, A., Shazeer, N., Parmar, N., Uszkoreit, J., Jones, L., Gomez, A. N., Kaiser, Ł., and Polosukhin, I. (2017). Attention is all you need. *Advances in neural information processing systems*, **30**.

Wang, D., Lin, H., Ma, Q., Bai, Y., and Qu, J. (2021). Manganese oxides in phragmites rhizosphere accelerates ammonia oxidation in constructed wetlands. *Water Research*, **205**, 117688.

Wang, D., Shang, J., Lin, H., Liang, J., Wang, C., Sun, Y., Bai, Y., and Qu, J. (2024). Identifying arg-carrying bacteriophages in a lake replenished by reclaimed water using deep learning techniques. *Water Research*, **248**, 120859.

Wang, J., Gou, Q.-y., Luo, G.-y., Hou, X., Liang, G., and Shi, M. (2022). Total rna sequencing of phlebotomus chinensis sandflies in china revealed viral, bacterial, and eukaryotic microbes potentially pathogenic to humans. *Emerging Microbes & Infections*, **11**(1), 2080–2092.
